# Emergency response and clinical treatment analysis of a mass wasp sting incident involving 38 tourists: A case report

**DOI:** 10.1097/MD.0000000000047126

**Published:** 2026-01-16

**Authors:** Yanmin Huang, Kangzhe Ruan, Wei Huang, Dihai Fang, Kai Li, Hailin Ruan

**Affiliations:** a Department of Emergency Medicine, The Fourth Hospital Affiliated Hospital of Guangxi Medical University/Liuzhou Worker’s Hospital, Liuzhou, Guangxi, China; b The Second Clinical Medical College, Wuhan University, Wuhan, Hubei Province, China; c The Fourth Affiliated Hospital of Guangxi Medical University, Liuzhou, Guangxi, China.

**Keywords:** follow-up, mass incident, prevention, tourists, treatment, wasp stings, wasps

## Abstract

**Rationale:**

Mass wasp sting incidents pose a significant public health threat, particularly to non-local tourists in scenic areas who face challenges such as unfamiliar healthcare systems and disrupted travel plans, which can compromise treatment compliance and outcomes. This study analyzes the emergency response and clinical management of such an incident to identify effective strategies for this specific population.

**Patient concerns:**

A total of 38 non-local tourists were stung by wasps at a scenic spot in Liuzhou, Guangxi. Primary patient concerns and clinical presentations included localized symptoms (redness, swelling, pain, and itching at sting sites) and systemic symptoms (dizziness, headache, anxiety, and rashes).

**Diagnoses:**

All patients were definitively diagnosed with wasp stings. Severity was classified according to the Chinese Expert Consensus on Standardized Diagnosis and Treatment of Wasp Stings, resulting in 33 mild cases (≤10 stings) and 5 moderate cases (>10 stings with systemic symptoms).

**Interventions:**

A multi-departmental emergency response was activated, involving the 120 dispatch center, fire department, and the hospital’s toxicology treatment center, achieving a median hospital arrival time of 35 minutes. Standardized in-hospital treatment included local wound management (iodine disinfection and wet compresses with dexamethasone and lidocaine), systemic therapy (IV dexamethasone, oral Jidesheng snake antivenom tablets, IM tetanus antitoxin), supportive care (hydration, oxygen), and proactive telephone follow-ups on days 3 and 7 post-discharge.

**Outcomes:**

All 38 patients recovered and were discharged. The median observation time was 8 hours for mild cases, while moderate cases were hospitalized for 2 days. Follow-up on day 3 revealed delayed-onset headaches or rashes in 8 patients (8/38), which completely resolved with intervention by day 7. No severe cases or fatalities occurred.

**Lessons:**

The successful outcome underscores the value of a seamless pre-hospital emergency response system and standardized clinical protocols (e.g., the “Four Duals” approach) for managing mass wasp stings. Proactive follow-up is crucial for non-local tourists to address compliance challenges and manage delayed symptoms effectively, suggesting that integrated emergency care systems and tailored patient management are key to improving outcomes in transient populations.

## 1. Introduction

Wasp stings represent acute-onset, clinically complex mass casualty incidents that may lead to life-threatening complications such as anaphylactic shock and multiple organ failure, posing significant threats to public health.^[1]^ Globally, with ecological environmental improvements and increased outdoor activities, the incidence of wasp stings has shown a marked upward trend,^[1–3]^ imposing substantial health and economic burdens on society. Domestic data indicate that sting-related mortality rates can reach 6%,^[4]^ highlighting its significant public health impact.

Such incidents frequently occur in mountainous scenic areas.^[5]^ The pathogenic mechanism involves venom components (including melittin, phospholipase A2, etc^[6]^) being injected through stingers, triggering a biphasic toxic reaction: early-stage local inflammation and allergic reactions,^[7]^ potentially progressing to hemolysis and acute kidney injury (most commonly occurring within 24 hours post-sting^[8]^) accompanied by electrolyte disturbances.^[9]^ This study suggests that in mass casualty management, timely response and cross-departmental collaboration are regarded as core strategies for reducing mortality.

Tourist populations face unique challenges in such incidents, including unfamiliarity with local healthcare systems, objective constraints from disrupted travel itineraries, barriers to cross-regional treatment continuity, and insufficient awareness of sting hazards. These factors collectively lead to reduced treatment compliance and shortened observation periods, thereby increasing complication risks. Existing literature has not systematically explored optimized treatment strategies for this special population. Therefore, studying treatment models and emergency response mechanisms for mass wasp sting incidents holds significant academic value while providing new perspectives for practical treatment.

Our hospital, as a municipal toxicology treatment center, treated 38 tourists stung by wasps on October 2, 2024, providing a critical research opportunity in this field. By analyzing this case’s emergency management processes and clinical intervention pathways, we aim to establish an evidence-based framework for tourist-oriented sting treatment.

## 2. Case report

### 2.1. Mass sting incident

On October 2, 2024, a mass sting incident occurred at a scenic spot in Liuzhou, Guangxi. A total of 38 cases were definitively diagnosed as wasp stings, representing the complete cohort for this case series. According to the severity classification in the Chinese Expert Consensus on Standardized Diagnosis and Treatment of Wasp Stings,^[10]^ cases were categorized as mild, moderate or severe (specific criteria shown in Table 1). This study included 33 mild cases and 5 moderate cases meeting these criteria.

**Table 1:** Grading criteria for wasp sting severity.

Severity	Number of stings	Local symptoms	Systemic symptoms	Organ involvement
Mild	<10	Redness, swelling, pain, itching; no necrotic lesions	None or only skin itching/erythema	No organ damage: ①Normal urine output, no hematuria/cola-colored urine ②Normal renal function (creatinine)
Moderate	10–30	Expanded area of swelling, possible local necrosis	Grade I–II allergic reaction: ①Generalized urticaria ②Nausea/vomiting ③Rhinorrhea, hoarseness, dyspnea	Single-organ injury: ①Oliguria (<0.5 mL/(kg·h) for > 6 h) ②Increased serum creatinine (≥0.3 mg/dL or ≥ 50%) ③SOFA score ≥ 2 (single system)
Severe	>30 (or grade III–IV allergic reaction)	Extensive necrosis, purulent lesions	Grade III–IV allergic reaction: ①Laryngeal edema/bronchospasm ②Shock/respiratory arrest ③Cardiac arrest	Multiple organ dysfunction: ①Cola-colored urine/hematuria ②Further reduced urine output (<0.5 mL/(kg·h) for > 12 h) ③Serum creatinine increase (200%–300%) ④ SOFA score ≥ 2 in ≥ 2 organ systems

SOFA = Sequential Organ Failure Assessment.

This study utilized retrospectively collected clinical data from de-identified patients. The Ethics Committee of Liuzhou Worker’s Hospital approved the protocol and waived the requirement for informed consent (approval no. KY2022343).

### 2.2. Population characteristics and clinical manifestations

All 38 cases were nonlocal tourists to the scenic area, including 18 from outside Guangxi and 20 from outside Liuzhou city proper; 20 males and 18 females (male: female ratio 1:0.9); age range 1–64 years (mean 25.39 ± 15.71 years). Sting sites were primarily exposed areas like head/face and upper limbs, all presenting local redness, swelling, pain, itching or rashes; systemic symptoms included dizziness, headache, anxiety and nervousness (specific clinical features shown in Table 2).

**Table 2:** Clinical manifestations of wasp sting patients.

Manifestations	Number of cases (n)	Percentage (%)	Manifestations	Number of cases (n)	Percentage (%)
Number of stings			Nervous system		
<10	33	86.84	Dizziness	31	81.58
>10	5	13.16	Restlessness	10	26.32
Sting sites			Anxiety	29	76.32
Head and face	38	100.00	Respiratory system		
Neck	33	86.84	Difficulty breathing	6	15.79
Chest and back	34	89.47	Circulatory system		
Upper limbs	35	92.10	Chest tightness and palpitations	15	39.47
Lower limbs	23	60.63	Digestive system		
Local manifestations			Nausea	8	21.05
Redness, swelling, pain	38	100.00	Urinary system		
Itching or rash	38	100.00	Tea-colored urine	5	13.16

### 2.3. Prehospital emergency response

**Figure 1.**
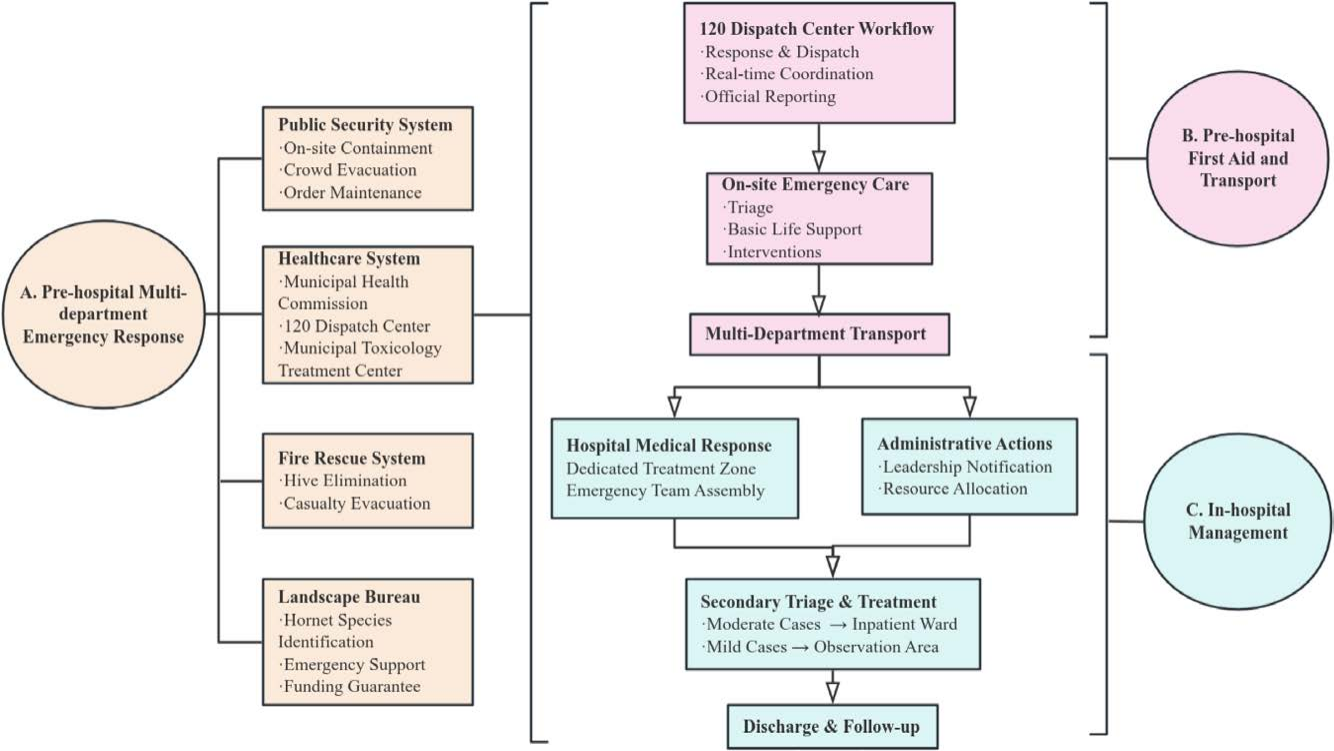
The flowchart illustrates the case management process, including prehospital multi-party coordinated rescue, graded disease management, and follow-up management.

At 17:32, the 120 dispatch center received emergency calls reporting multiple tourists stung by wasps at a scenic spot and immediately initiated response, coordinating emergency resources from various stations while linking with the toxicology treatment center and reporting to the Municipal Health Commission. Police officers secured the scene and maintained order; firefighters guided tourists to move away from the area to safety. Ambulances were dispatched at 17:34, rapidly reaching the scene. En route, medical personnel provided telephone guidance to on-site personnel regarding keeping patients calm and avoiding scratching wounds. Upon arrival, medical staff immediately conducted injury assessments (flowchart shown in Fig. 1).

During on-site management and transport, close monitoring of condition changes and vital signs was maintained, with oxygen administration, IV access established for some patients, and psychological reassurance provided to prevent anaphylactic shock and alleviate symptoms like breathing difficulties, restlessness and anxiety. Safety belts were secured to ensure safe transport. The receiving hospital was notified in advance to activate emergency green channels. Ambulances from multiple stations and police vehicles jointly transported patients to our hospital, achieving a median arrival time of 35 minutes post-sting (range 28–42 minutes).

### 2.4. In-hospital clinical treatment

The emergency department simultaneously activated mass incident protocols, with hospital leadership establishing a command group: dedicated centralized treatment wards and observation areas were prepared, with EICU (Emergency Intensive Care Unit) and dialysis units on standby; the pharmacy department prepared emergency medications including dexamethasone and Jidesheng snake antivenom; the logistics department ensured blood purification equipment readiness. After prehospital to in-hospital handoff, injury assessments and triage were completed: 33 mild cases were placed in emergency observation areas while 5 moderate cases were admitted to inpatient wards. All patients were treated under the “life-saving first, payment later” principle with continuous green channel access. Standardized treatment protocols per guidelines included:

Local management: Iodine disinfection followed by continuous wet compresses with a mixed solution of dexamethasone (10 mg) + 2% lidocaine 100 mg + normal saline 100 mL.

Systemic therapy: IV (Intravenous) dexamethasone 10 mg (diluted in 250 mL normal saline), oral Jidesheng snake antivenom tablets (initial dose 20 tablets, followed by 6 tablets every 6 hours), IM (Intramuscular) tetanus antitoxin 1500 IU.

Supportive care: crystalloid hydration (initial 1000–1500 mL, adjusted based on blood pressure/urine output) and continuous oxygen therapy.

Enhanced monitoring: blood pressure and urine output assessments every 2 hours, liver/kidney function and electrolyte tests within 6 hours, concurrent anxiety counseling and prognosis education.

### 2.5. Discharge instructions and follow-up

Patients were advised postdischarge to increase fluid intake and continue prescribed oral Jidesheng antivenom and prednisone. They were instructed to seek immediate medical attention if experiencing intractable itching, breathing difficulties, oliguria or anuria to prevent serious complications. Two follow-ups were conducted on days 3 and 7 postdischarge.

### 2.6. Clinical outcomes

Mild cases had a median observation period of 8 hours before discharge, while moderate cases were hospitalized for 2 days. Day 3 follow-up identified 8 mild cases with persistent local pain or rashes, which resolved within 24 hours after topical steroid treatment. By day 7 follow-up, all 38 patients met full recovery criteria: resolution of local redness/swelling/pain/itching and systemic dizziness/chest discomfort symptoms.

## 3. Discussion

### 3.1. Value of efficient emergency response and systemic support

Mass casualty incidents represent important triggers of public health events,^[11]^ posing serious threats to public health and safety that draw widespread social attention.^[12]^ This wasp sting incident occurred at a scenic spot during National Day holiday, involving numerous nonlocal tourists with significant social impact. Under government leadership, leveraging established urban emergency systems (integrating medical institutions, emergency dispatch centers, public security, fire departments and landscape management), rapid response and efficient coordinated treatment were achieved. In this study, all patients reached the hospital within a median time of just 35 minutes, ensuring interventions within the critical “golden 6-hour” window, effectively preventing incident escalation and minimizing negative social consequences. This efficient response not only benefited from multi-department coordination mechanisms but also highlighted the crucial value of establishing regional toxicology treatment centers. Through implementing standardized treatment protocols, integrating prehospital emergency-inpatient emergency-critical care “treatment chains,” and providing advanced life support capabilities like blood purification, these centers offer powerful technical support for managing poisoning-related public health emergencies. As one of the first municipal-level toxicology centers in the region, our facility achieved seamless continuity of care from prehospital to postdischarge follow-up for poisoned patients during this incident, ensuring uninterrupted treatment for critical cases and directly improving treatment timeliness and success rates. Currently, the development of national and provincial toxicology center networks is systematically enhancing China’s capacity for poison incident prevention and treatment.

### 3.2. Characteristics of wasp sting complications

Consistent with previous studies,^[13]^ our observations also confirmed that most wasp stings manifest as local reactions (e.g., redness/swelling, pain, itching). However, notably, all patients in this series exhibited varying degrees of allergic reactions (e.g., rashes, itching), with 8 cases (21.1%) still showing symptoms (dizziness, headaches, rashes, etc) during day 3 follow-up after discharge, suggesting possibilities of delayed allergic reactions or incomplete recovery. Wasp venom contains complex components (e.g., antigen-5, phospholipase A, hyaluronidase, etc^[14]^) that not only cause local inflammation and allergic reactions^[15]^ but may also lead to multi-organ damage or even death in severe cases.^[16]^ While sting-related allergic reactions are typically immediate,^[10]^ delayed reactions (e.g., urticaria, arthralgia)^[17]^ also warrant attention, with our follow-up data providing clinical evidence for this phenomenon.

### 3.3. Clinical value of the “four duals” therapeutic approach

The key to this study’s success lay in implementing standardized emergency strategies – the “four duals” approach10 – within the “golden 6-hour” window. Benefiting from rapid hospital arrival (median 35 minutes), all patients received systematic treatment during the critical period:

“Dual early” (early assessment and early intervention): quickly stabilized emotions, managed wounds to prevent infection, and alleviated local symptoms.

“Dual anti” (antiallergy and antishock): addressed allergic manifestations in all patients through prompt prednisone administration.

“Dual hormones” (glucocorticoids and epinephrine): epinephrine as first-line for anaphylactic and glucocorticoids for sustained anti-inflammatory effect.

“Dual hydration/alkalization” (hydration and alkalization): adequate IV hydration maintained perfusion and urine output,^[18]^ promoting toxin elimination – the cornerstone for preventing venom-induced rhabdomyolysis and hemolytic acute kidney injury.

The effective execution of the “four duals” approach relied not only on clear clinical pathways but also benefited from the aforementioned efficient emergency systems and standardized toxicology center protocols, demonstrating synergistic value between clinical interventions and system optimization. Additionally, the Jidesheng snake antivenom used in this case has not yet received international recommendation. In China, some toxicology centers empirically employ it in practice based on the presumption of potential cross-neutralization effects against phospholipase A2.

### 3.4. Challenges with nonlocal tourists and targeted interventions

A special challenge in this study was that all patients were nonlocal tourists. Tourists often exhibit reduced compliance with local treatment due to unfamiliarity with regional healthcare systems, disrupted travel plans, inconvenience of cross-regional care, and insufficient awareness. In our series, 8 patients still had symptoms during initial follow-up (day 3) due to premature discharge or inadequate observation. This directly illustrates how reduced compliance may lead to incomplete treatment, increasing risks of delayed reactions or prolonged recovery. Our data suggest that follow-up, as a critical extension of medical services, assumes particular importance for such populations, playing special roles in ensuring treatment efficacy and timely identification/management of potential risks. Healthcare providers should maintain heightened awareness for these special patients, strengthening follow-up commitment and responsibility while optimizing follow-up methods and content (including telemedicine consultations). Through such measures, treatment outcomes and satisfaction for nonlocal tourist patients can be further improved.

### 3.5. Multi-stakeholder collaboration in prevention

This incident also sounded an alarm regarding travel safety. Currently, no specific antidotes exist for wasp stings clinically, making prevention paramount.^[19]^ Tourists in lush scenic areas should enhance personal protection by^[20]^: avoiding exposed food (especially meat and sweet beverages); refraining from perfume use^[21]^; high allergy-risk individuals must carry epinephrine auto-injectors and use them immediately if experiencing throat tightness or breathing difficulties^[22]^; remaining calm and slowly moving away when encountering wasps, covering exposed areas with clothing if attacked by swarms.^[23]^ Beyond individual precautions, systematic prevention is essential: governments and scenic areas should strengthen public education; scenic management departments should collaborate with fire and forestry services for regular risk assessments, environmental cleanup, and warning signage; staff emergency training (including first aid skills) should be enhanced; during incidents, rapid crowd evacuation, emergency reporting and professional nest handling are critical.

### 3.6. Study limitations and future directions

This study has limitations including its single-center retrospective design and relatively small sample size (n = 38), with all subjects being a specific tourist cohort, potentially limiting generalizability (e.g., to local residents). The absence of control groups hinders precise quantification of individual contributions from specific “four duals” components (e.g., anti-allergy therapy, hydration). Additionally, high patient mobility created follow-up difficulties, with telephone self-reports carrying recall bias risks, while potential loss to follow-up may affect delayed allergy reaction rate estimates. Retrospective data collection might also reduce accuracy of key metrics (e.g., exact sting counts, venom load measurements). Future studies should adopt multicenter prospective designs incorporating larger, more diverse cohorts with control groups to validate intervention effects; exploring integrated electronic health records (EHR) and wearable technologies to optimize mobile population data management is recommended.

## 4. Conclusion

The emergency management of this mass wasp sting incident demonstrated that government-led multi-department coordination combined with standardized treatment protocols (e.g., the “four duals” approach) at regional toxicology centers can significantly reduce time-to-treatment (median 35 minutes), ensure effective critical window interventions, and prevent severe outcomes. The study also revealed delayed reaction risks (21.1%) in nonlocal tourists due to compliance issues, necessitating tailored management strategies like remote follow-up systems. However, the study’s single-center design, small sample size (n = 38) and retrospective data collection limit generalizability, warranting future multicenter prospective studies incorporating digital fowllow-up platforms (e.g., WeChat mini-app, online symptom diary) to overcome the loss to follow-up observed in mobile tourist populations.

## Author contributions

**Investigation:** Dihai Fang, Kai Li.

**Resources:** Wei Huang.

**Writing – original draft:** Yanmin Huang, Kangzhe Ruan.

**Writing – review & editing:** Hailin Ruan.
